# Zygomycosis in Immunocompromised non-Haematological Patients

**DOI:** 10.4084/MJHID.2011.012

**Published:** 2011-03-15

**Authors:** George Petrikkos, Miranda Drogari-Apiranthitou

**Affiliations:** 4st Dept. of Internal Medicine, School of Medicine, National and Kapodistrian University of Athens, “ATTIKON” Hospital, RIMINI 1 – Haidari, Athens - 12464. Greece

## Abstract

Zygomycoses caused by fungi of the mucorales order (mucormycoses) are emerging fungal diseases with a high fatality rate. The most important risk factors include neutropenia or functional neutropenia, diabetic ketoacidosis, iron overload, major trauma, prolonged use of corticosteroids, illicit intravenous drug (ID) use, neonatal prematurity, malnourishment, and maybe a previous exposure to antifungal agents with no activity against zygomycetes, such as voriconazole and echinocandins.

A high index of suspicion is crucial for the diagnosis, as prompt and appropriate management can considerably reduce morbidity and mortality. Suspicion index can be increased through recognition of the differential patterns of clinical presentation. In the non- haematological immunocompromised patients, mucormycosis can manifest in various clinical forms, depending on the underlying condition: mostly as rhino-orbital or rhino-cerebral in diabetes patients, pulmonary infection in patients with malignancy or solid organ transplantation, disseminated infection in iron overloaded or deferoxamine treated patients, cerebral - with no sinus involvement - in ID users, gastrointestinal in premature infants or malnourishment, and cutaneous after direct inoculation in immunocompetent individuals with trauma or burns.

Treating a patient’s underlying medical condition and reducing immunosuppression are essential to therapy. Rapid correction of metabolic abnormalities is mandatory in cases such as uncontrolled diabetes, and corticosteroids or other immunosuppressive drugs should be discontinued where feasible. AmphotericinB or its newer and less toxic lipid formulations are the drugs of choice regarding antifungal chemotherapy, while extensive surgical debridement is essential to reduce infected and necrotic tissue. A high number of cases could be prevented through measures including diabetes control programmes and proper pre- and post-surgical hygiene.

## Introduction:

The term ‘Zygomycosis’ refers to a group of rare infections caused by hyaline filamentous fungi belonging to the class of Zygomycetes. This class is subdivided in two orders, Mucorales and Entomophthorales, both involved in human disease.^1^

Mucorales are saprobiotic organisms ubiquitous in nature; some members of this order are weak plant parasites or plant pathogens and opportunistic pathogens for man.^2^ The vast majority of human zygomycotic disease is caused by genera of the Mucorales order, therefore, the term ‘mucormycosis’ is used interchangeably with the term ‘zygomycosis’ (the term ‘phycomycosis’ has also been used in the past). The most common genera confirmed in human disease are: *Rhizopus*, *Mucor*, *Lichtheimia* (formerly known as *Absidia corymbifera* or *Mycocladus*), *Rhizomucor*, *Apophysomyces*, *Saksenaea*, and *Cunninghamella*.^1^

For Mucorales the portals of entry in the human body are the respiratory tract through inhalation of fungal spores, the skin and less frequently the gut. They grow rapidly and produce wide hyaline, aseptate or poorly septated ribbon-like hyphae in tissues. They invade blood vessels and cause thrombosis and necrosis of the infected tissues. Disease in humans is limited to severely immunocompromised individuals, those with diabetic ketoacidosis, or those with burns or trauma. Infection usually progresses rapidly and the case fatality rate is very high. Depending on the underlying condition and the portal of entry they can cause rhinocerebral, pulmonary, cutaneous, gastrointestinal or even disseminated infection.^1^

Human infections caused by Entomophthorales, which are mainly insect pathogens, constitute a completely different clinical entity (entomopfthoramycoses). Genera involved in human disease are *Conidiobolus* and *Basidiobolus*. They affect mainly immunocompetent individuals, are prevalent in the tropics and do not disseminate.^3^ In recent years the geographic distribution of basidiobolomycosis expanded and is reported also in immunocompromised hosts involving more tissues, but these cases are extremely rare.^4^,^5^ Therefore, the present review focuses only on the zygomycotic infections caused by mucorales.

The most important risk factors for mucormycosis include prolonged neutropenia, diabetic ketoacidosis, iron overload and major trauma. Other important predisposing factors of mucormycosis include prolonged use of corticosteroids, illicit intravenous drug use, neonatal prematurity, malnourishment and broad-spectrum antimicrobial agents, as well as antifungal agents with no activity against zygomycetes, such as voriconazole and caspofungin.^6^,^7^ The same may also be true for the other echinocandins, but data are lacking as they are not as widely used as caspofungin. Risk factors, mechanisms leading to mucormycosis and clinical presentation forms are illustrated in Table 1.

The recent years, mucormycosis incidence is seemingly increasing, particularly in patients with haematological malignancies or haematopoietic stem cell transplantation, at least in western countries.^8^,^9^,^10^ However, accurate estimation of the incidence is a challenge. Data from a global registry are not available yet and moreover there are difficulties in collecting well defined denominator data. Much information has been derived from the first most comprehensive study by Roden et al. 2005,^6^ in which all data on zygomycosis recorded in the English language literature ever since the first report by Platauf in 1885,^11^ were analyzed. More recent data from individual countries and institutions have also been reported^12^–^17^ and percentages of clinical cases according to underlying condition are presented in Table 2.

### Underlying Conditions

#### Diabetes – Ketoacidosis:

Diabetes mellitus as a predisposing factor has been reported in 36–88% of all mucormycosis cases.^6^,^18^–^20^ More susceptible are patients with uncontrolled hyperglycemia, particularly those with ketoacidosis.^21^,^22^,^23^ Mucormycosis can also be observed in metabolically controlled diabetic patients^24^ and was found to be the first clinical manifestation of some patients with undiagnosed diabetes mellitus.^25^ Type 1, type 2, and secondary diabetes mellitus have all been reported as risk factors in patients with mucormycosis.^26^

In the normal host protection from mucormycosis is provided by macrophages. They prevent the initiation of infection by phagocytosis and oxidative killing of the fungal spores. In case infection is established, neutrophils are activated and play a pivotal role in fungal killing. Activation happens in four phases:

- neutrophils are chemotactically attracted to the hyphae,
- they attach and
- spread on the hyphae and finally,
- using their oxidative cytotoxic system, neutrophils damage and kill the fungal elements without accompanying phagocytosis.

In diabetes, each of the four phases of neutrophil activation is impaired.^27^ Hyperglycemia per se does not seem to play a major role in the pathogenesis of mucormycosis. Chemotaxis, the phagocytic functions (adherence and spreading), and finally the oxidative burst are all inhibited in the ketoacidotic state, essentially inducing functional neutropenia.^26^–^29^

The most common clinical presentation of mucormycosis in patients with diabetes mellitus is sinus disease (66%).^6^ Diabetes mellitus was recognized as a major risk factor for developing rhinocerebral mucormycosis very early on. The publication by Gregory et al. in 1943 on a case of uncontrolled diabetes was the first to describe fulminant rhinocerebral mucormycosis.^30^

Disease in diabetics can also present as pulmonary mucormycosis, a clinical presentation more common in neutropenic patients with malignancies or HSCT.^6^

The epidemiology in diabetic patients is variable. Roden et al.^6^ showed that diabetic patients represented 36% of the 929 reported cases. They also reported a decreased incidence of mucormycosis in diabetic patients over time. This is in contrast to the increased prevalence of diabetes in the world.^31^ The role of statins, used at least in the western world, has been speculated in an effort to explain this phenomenon.^32^,^33^ In France, however, a population- based study of medical records of mucormycosis cases reported from 1997 to 2006 showed a 9% yearly increase in the annual incidence rate in the diabetic population.^12^ Another retrospective study from the USA, reviewing cases with rhino-orbital-cerebral mucormycosis from two teaching tertiary-care hospitals revealed that 83% of cases were observed in diabetic patients and more importantly, 41% of them had no known history of their condition.^34^ A striking finding in this study was that 56% of the patients were of Hispanic origin, a much larger proportion than that in the general population (≈25% expected incidence of Hispanic ethnicity based on demographic characteristics at the study hospitals). Data from a tertiary-care centre in India^35^ were also overwhelming, as 73.6% of cases were observed in patients with uncontrolled diabetes and in 42.7% of these cases diabetes was diagnosed for the first time. These findings also underline the association between diabetes and socioeconomic status. Low income individuals have the tendency not to seek medical attention as healthcare is unaffordable to them, unless complications manifest. Almost 80% of type 2 diabetes deaths occur in low- and middle-income countries (WHO Fact sheet: Diabetes, ref.^36^). With diabetes control programs in place, also many cases of mucormycosis could be prevented.

The overall mortality of diabetic patients with mucormycosis who undergo treatment is approximately 44%, and this is lower compared to other immunocompromising conditions.^6^ This can be explained by the relatively easy management of acute complications of diabetes compared to the management of other conditions. This percentage can be further decreased with improved management.^37^

Besides diabetic ketoacidosis, chronic metabolic acidosis due to other causes, such as chronic renal failure with uremia,^38^ chronic salicylate poisoning,^39^ and methylmalonicaciduria,^40^ has also been reported as a risk factor for mucormycosis.

#### Iron overload and Deferoxamine (DFO) iron/aluminium chelation therapy:

Many microorganisms require iron to grow, and conditions of iron excess have been known to predispose to infection with certain bacteria.^41^,^42^ Iron acquisition is a critical step in the pathogenetic mechanism of mucormycosis.^43^

Therapy with DFO, an iron chelator used for the treatment of iron and/or aluminum overload in dialysis patients, was found paradoxically to be a risk factor for angioinvasive mucormycosis.^44^,^45^ Although the first dialysis patient with mucormycosis, reported in 1979 by Gluskin *et al*,^46^ was not noted to be receiving DFO, subsequent cases of mucormycosis in patients undergoing dialysis have been in those receiving DFO for aluminum or iron excess.^45^ A report of an international registry^47^ showed that 78% of dialysis patients with mucormycosis were being treated with DFO. In addition to patients with renal failure, patients with hematologic disorders, all of whom were treated with DFO, have been reported with mucormycosis.^45^ Subsequent research showed that DFO can act as a siderophore for Mucorales: DFO forms a Fe-DFO complex with iron, which then binds to unidentified receptors on the surface of the zygomycetes. The iron is subsequently liberated by the fungus and is likely transported into the fungal cell by a high-affinity iron permease (rFTR1).^43^ The increased sensitivity of dialysis patients to DFO-related mucormycosis is explained by the pharmacokinetic changes during uremia, which lead to a prolonged accumulation of Fe-DFO after DFO administration.^48^

Besides DFO, iron overload per se, either transfusional or due to dyserythropoiesis has also been recognized as a risk factor for mucormycosis.^49^–^51^ In vitro and in vivo studies have shown that iron and DFO can enhance both growth and pathogenicity of *Rhizopus spp*. In a study reviewing a series of five cases of invasive mucormycosis among 263 allogeneic bone marrow transplant recipients,^51^ the association between severe iron overload and mucormycosis was demonstrated. The mean values of serum ferritin level, transferrin saturation, and number of transfused units of erythrocytes in the study group were significantly higher compared with the matched control group.

Patients with diabetic ketoacidosis have elevated levels of available serum iron, likely due to the release of iron from binding proteins in the presence of low pH. Iron is then internalized by the zygomycetes with the help of copper oxidase (Cu-oxidase) and the high affinity iron permease rFTR1. Therefore, the increased susceptibility of patients with diabetic ketoacidosis to mucormycosis is likely due, at least in part, to an elevation in available serum iron during diabetic ketoacidosis following proton-mediated dissociation of iron from transferrin.^43^

The most common presentation of mucormycosis in patients receiving DFO appears to be the disseminated form (44%) and is associated with high mortality, reaching 80%.^6^,^47^

In contrast to DFO, two other iron chelators, deferiprone and deferasirox, do not supply iron to the fungus. This could be either because of their different structures and smaller size, rendering them inaccessible to fungal iron uptake systems, or because they share higher affinity constants for iron.^52^ Deferiprone and deferasirox were shown to have fungicidal activity against Zygomycetes in vitro. Further, both iron chelators were shown to effectively treat mucormycosis in animal models, and one has been successfully used as salvage therapy for a patient with rhinocerebral mucormycosis.^43^

#### Solid organ malignancies and solid organ transplantation (SOT):

The incidence of mucormycosis in transplant recipients, including both hematopoietic stem cell transplant (HSCT) and solid organ transplant (SOT) patients has increased. Although global surveys of the incidence of mucormycosis are still lacking, data from individual countries and institutions show that mucormycosis in SOT is rare, but seems to be a concern because of the high mortality rate.

The estimated incidence ranges from 0.4–16%.^6^,^12^,^52^,^53^ Data from the Transplant Associated Infection Surveillance Network (TRANSNET) showed that among 1063 solid organ transplant recipients mucormycosis represented 2% of invasive fungal infections. Neutropenia predisposing to mucormycosis^6^ was notably absent in SOT recipients as were acidosis with or without hyperglycemia and use of DFO. In contrast, all patients were receiving immunosuppression and the overwhelming majority was on corticosteroids. Furthermore, dissemination to distant organs occurred more frequently after rejection and its treatment.

The lung seems to be the most common site of infection in SOT recipients with mucormycosis.^6^,^54^ Dissemination occurred preferentially to cutaneous and soft tissues, and not the brain. *Mycocladus* (*Lichtheimia*) *corymbifer* as causative pathogen appeared to be associated with a higher risk for dissemination in SOT with pulmonary mucormycosis in one study.^55^

In a matched case-controlled study^54^ SOT recipients with mucormycosis were prospectively studied. Renal failure, diabetes mellitus, and prior voriconazole and/or caspofungin use were associated with a higher risk, whereas tacrolimus was associated with a lower risk of mucormycosis. Liver transplant recipients were more likely to have disseminated disease and developed mucormycosis earlier after transplantation than did other SOT recipients (median, 0.8 versus 5.7 months).

#### HIV/AIDS - Intravenous drug abuse:

Mucormycosis in HIV/AIDS patients is very rare. Antinori et al.^56^ in a large retrospective study of 1630 autopsies of patients who died of AIDS during 1984 through 2002, found only 2 cases with mucormycosis. However, it can be the presenting opportunistic infection in AIDS.^57^

Recently, human immunodeficiency virus (HIV) infection has been recognized as a risk factor for mucormycosis, but most cases in HIV-infected patients are also associated with intravenous drug abuse.^58^,^59^,^60^,^61^ Cerebral mucormycosis with basal ganglia involvement is the usual clinical presentation in such cases, regardless of previous exposure to HIV.^62^ The disease may develop insidiously or may progress rapidly with a fulminant course.

Endocarditis as well as the rhinocerebral form can also occur. Merchant et al.^63^ reported on a patient with rhinocerebral mucormycosis and also reviewed another 6 cases reported in the literature. It is of note that 3 of them had hyperglycemia or diabetic ketoacidosis.

Mucormycosis in the HIV+ can also present in the kidneys, the skin, the gastrointestinal tract, the respiratory tract, or may be disseminated.^64^–^75^ How the fungus enters the body is not very clear. The fact that most of the infections occur at sites remote from the needle stick however, suggest that most probably this happens through spores contained in the illicit drugs.^1^

#### Corticosteroids/Rheumatic diseases:

Corticosteroid therapy is another primary risk factor that enhances a patient’s susceptibility to mucormycosis by causing either defects in macrophages and neutrophils or steroid-induced diabetes.^1^

Corticosteroids have a wide range of complex immunosuppressive effects, mainly by affecting cellular immunity, and increase host susceptibility to invasive fungal infections.^76^ The mechanism by which the corticosteroids enhance susceptibility to developing mucormycosis is probably twofold. First, steroids suppress the normal inflammatory cell response that would otherwise occur, and second, they may induce a diabetic state.^77^ In a recent study only 21% of patients with corticosteroid induced diabetes were receiving medication for diabetes.^34^

Of the few cases of mucormycosis in patients with lupus erythematosus (SLE) reported in English language literature^78^–^93^ it appears that disease can present with any clinical form, but disseminated mucormycosis is usual in this group of patients. Mok et al.^78^ reviewed all SLE cases published from 1970 through 2002 and reported a very high overall mortality of mucormycosis (88%). Additional predisposing factors for opportunistic infection included hypocomplementemia, nephrotic syndrome, uremia, leukopenia, and diabetes mellitus. The diagnosis often was made only at autopsy (63%). The cutaneous form appeared to have the best prognosis with combined medical and surgical treatment.^78^

Mucormycosis in other autoimmune diseases are scarce, but in cases such as in Wegener’s granulomatosis it may mimic disease relapse and remain underdiagnosed.^94^

#### No underlying disease:

A considerable proportion of patients with mucormycosis have no apparent immune deficiency.^6^ These patients have usually primary mucormycosis of the skin associated with burns, trauma, insect bites, tattooing and also complicating surgical wounds and catheter insertion sites. Recently, Skiada and Petrikkos reviewed 67 cases of cutaneous mucormycosis in English language literature, from 2004–2008.^95^ Most of the patients had an underlying immunocompromising condition such as haematologic malignancy, diabetes, or solid organ transplantation, but the immunocompetent represented a large proportion (40%).

Infection usually occurs through direct inoculation. It can be very invasive locally, penetrating the cutaneous and subcutaneous tissues into the adjacent fat, muscle, fascia and bone. In rare cases it can even disseminate to deep organs. Road traffic accidents, crush injuries, but also minor traumas such as those caused by plant thorns or insect bites, can lead to traumatic implantation of contaminated soil and subsequent mucormycosis. Major burn injury is another well described cause of cutaneous mucormycosis. An increased risk for developing mucormycosis in burn patients is the development of a condition called “burned stressed pseudodiabetes”, which is marked by hyperglycemia and glycosuria.^96^,^97^

In a new PubMed search from 2008 – 2010, we found 27 additional cases of cutaneous mucormycosis occurring in non immunocompromised hosts.^98^–^110^ Risk factors in these cases included soil contaminated wounds, insect bites, burns, but the majority of cases (N=12) were iatrogenic, either after surgery or at injection sites. Infections with Mucorales occurring in nosocomial settings have been repeatedly reported and have even caused small hospital outbreaks in the USA, the UK and in Europe.^111^ Sources of infection included contaminated bandages, Elastopad adhesive dressings, wooden tongue depressors and non-sterile karaya ostomy bags.

The clinical presentation is variable. The clinical signs initially are non specific, consisting of erythema and induration. Eventually the lesions may form blisters, pustules, or necrotic ulcerations. Cotton wool-like material may also be observed on the margins of the wound. The lesions may mimic pyoderma gangrenosum, bacterial synergistic gangrene or infections produced by *Pseudomonas*, *Aspergillus*, *Histoplasma* etc. Biopsy specimens for histology and cultures are necessary to establish diagnosis.

Strains involved are usually *Rhizopus spp*. (the majority), *Lichtheimia corymbifera* (syn. *Absidia, Mycocladus*), *Apohysomyces elegans*, *Mucor spp.*, *Cunninghamella bertholetiae* and *Saksenaea vasiformis*. In the cases reported after 2008 the cultured species were *R. arrhizus* in 3 cases,^99^,^101^ *Lichtheimia spp.*in 5,^98^,^100^,^107^,^109^ *A. elegans* in 4,^98^ *S. vasiformis* in 3,^98^,^102^,^110^ and *Rhizomucor variabilis* in 7.^103^–^104^ The later, a non-thermophile species of the genus *Rhizomucor*, in contrast to other mucorales can cause large but slowly progressing opportunistic infections of the skin in immune-competent individuals. These fungi seem to have an increasing incidence in farmers from China.

The prognosis of mucormycosis in the immunocompetent is better than that in other patient groups but mortality remains high depending on the site, extension of infection and time of initiation of antifungal therapy. Iatrogenic cutaneous mucormycosis could easily be prevented with proper preoperative preparation and postoperative dressing of the surgical sites.

## Diagnosis:

The clinical diagnosis of mucormycosis among non haematological immunocompromised patients is notoriously difficult because of the similarity with aspergillosis or other mycoses caused by filamentous fungi. Diagnosis is usually delayed, as the majority of the physicians are not so much familiar with the disease. The leading signs and symptoms of mucormycosis are presented in Table 3.

When clinical presentation may suggest mucormycosis, imaging techniques are helpful, but cultures and histopathology are required for confirmation.

In case of rhinocerebral involvement, CT and particularly MRI are helpful in enabling an early detection of orbital, sinus, meningeal, bone, and cerebral lesions as well as intracranial vascular occlusion even before clinical signs develop. In pulmonary mucormycosis, lung biopsies (endoscopic, CT-guided or surgical) should be performed, depending on the radiological findings obtained by CT scans. CT guided percutaneous lung biopsy has been found highly efficient to early differentiate aspergillosis from mucormycosis in haematological patients. Whatever the initial clinical site involved, a sinus and chest CT are mandatory in addition to brain imaging. This is of major importance for the indicated therapeutic approach.^112^

The laboratory diagnosis of mucormycosis is challenging and requires expertise and proper sampling. Proper clinical samples include scrapings and aspirates from sinuses, nasal discharges, BAL, needle biopsies from pulmonary lesions, skin scrapings from cutaneous lesions and biopsy tissues. Zygomycetes are almost never isolated from blood and very rarely isolated from cultures of cerebrospinal fluid, sputum, urine or faeces, or swabs of infected areas.^113^

The significance of the isolates grown in cultures may be doubtful, especially when grown from samples of non sterile sites, as they are commonly encountered as contaminants. Histopathologic evidence of fungal invasion of tissue is required to confirm clinical or radiological diagnosis and/or reliability of culture.

The direct microscopic examination of biopsy material in 10%–20% KOH and/or calcofluor-white wet mount shows characteristic broad (6–15 μm in diameter), thin walled, mostly aseptate, ribbon-like hyaline hyphae with almost right –angle branching at irregular intervals. In tissue, Zygomycetes hyphae can be distinguished from the regularly septated hyphae of more common opportunistic molds such as *Aspergillus spp*. Histopathologic sections occasionally show folded, twisted, and compressed hyphae, which may be mistaken for septated hyphae. Sporangia, the reproductive hyphal structures that contain spores, are seen rarely in tissue in patients infected with zygomycetes. These hyphae are poorly stained with PAS and Gram stains but are very well seen by H&E, GMS, or Periodic-acid Schiff stain. Hyphae may be observed within necrotic tissue and signs of angioinvasion and infarction; neutrophilic infiltrates or granuloma formation may be present in patients who are not granulocytopenic or with more chronic infection, respectively.^114^

Identification of zygomycetes at the genus and species levels requires culture studies, because all members of this group are morphologically similar in tissue. Poor recovery of Zygomycetes may reflect limited septation of the hyphae, making the fungi more liable to damage resulting from tissue excessive grinding. Samples should be kept at room temperature until culture plating, as these fungi may not survive refrigerator temperatures, and excessive grinding should be avoided. The zygomycetes can be easily grown on conventional media like SDA with antibiotics, at temperatures 25° C to 37°C, without cycloheximide as this substance is inhibitory to most of them except *A. elegans*. If all other media fail, sterile bread without preservatives in a test tube may recover zygomycetes from clinical samples, since these fungi are commonly associated with bread.^115^ The rapidly growing mycelia are described as fibrous or cotton-candy.

There are no reliable serologic or skin tests for mucormycosis and the recently introduced antigen tests for Aspergillus (galactomannan) and other fungal species (b-D-glucan) do not detect Zygomycetes because of the limited amount of galactomannan and glucan in their cell walls.^116^

When cultures are negative, molecular identification of zygomycetes from fresh frozen or paraffin embedded tissue can help to confirm diagnosis and identify the fungus to the genus and species level. Different techniques have been reported: DNA probes targeting the ribosomal 18S subunit, ITS1 sequencing after PCR with pan-fungal primers, 18S-targeted semi-nested PCR and real-time PCR targeting cytochrome b gene.^117^–^122^ The identification to the species level of a strain isolated in culture and the identification of a zygomycete in tissue by PCR make the diagnosis much easier. However, at present, there is no standardized method available. Nevertheless, problems remain, since cultures are positive in only 50% of cases^6^ and tissue for examination is not always available.

Sequencing of PCR products leads to a presumptive identification of the Zygomycetes, which provides some guidance in selecting antifungal therapy that would not have been available using histopathology alone. Molecular typing is most useful for characterizing zygomycete epidemiology in the setting of a cluster of nosocomial cases, outbreaks, or pseudo-outbreaks, in order to rule out clonal spread or a common infecting source.

## Treatment:

The prognosis of patients with zygomycosis due to Mucorales without treatment is very poor approaching 100%, depending on the underlying condition and form of mucormycosis.^123^ Given the rapid development of the disease, early diagnosis and immediate initiation of treatment is critical for a favourable outcome.^124^

The therapeutic approach to mucormycosis is multimodal, with an equally important three-point strategy that includes (a) antifungal therapy, (b) surgical debridement and (c) correction of the underlying condition predisposing the patient to the disease. Moreover, management of co-morbid factors and adjunctive treatments may improve host response.

Because of the relative rarity of mucormycosis, prospective, comparative studies of antifungal agents and strategies have not been conducted. Therefore, the management of mucormycosis is so far based on the results of case series and case reports, animal model studies and in vitro susceptibility data.

The most frequently used antifungal agents to treat mucormycosis are listed in Table 4.

Amphotericin B has the most significant in-vitro activity against Zygomycetes and has been successfully used to treat mucormycosis for many years. Amphotericin B deoxycholate (d-AmB) is the only antifungal agent that has been approved by the US Food and Drug Administration for primary treatment of mucormycosis. However, this formulation has significant toxicity. The newer lipid formulations of amphotericin B that include liposomal AmB (L-AmB), AmB lipid complex (ABLC), and AmB colloidal dispersion (ABCD) are less nephrotoxic and have almost completely replaced amphotericin B deoxycholate in the recent years. It seems reasonable to recommend either L-AmB or ABLC as first-line treatment for mucormycosis.^125^–^128^ The optimal daily dose, as well as the length of treatment, are both undefined yet. Starting dosages of 5–7.5 mg/kg/day for L-AMB and of 5mg/kg/day for ABLC, respectively, are commonly used for adults and children.^129^

Of the azoles only posaconazole has in vitro activity against Zygomycetes and has been shown to be active as salvage therapy.^130^,^131^ Some physicians are using the combination of posaconazole with amphotericin B, but the efficiency of this approach has not been proven in clinical trials.

Zygomycetes are resistant to caspofungin and 5-flucytosine in vitro.^132^,^133^ Itraconazole may have some activity against certain strains of *Rhizomucor* and *Lichtheimia*^134^ and could be an option especially in cases of cutaneous infection. However, despite rare case reports,^135^–^138^ data are insufficient to support its use as monotherapy of mucormycosis in clinical practice.

There is some evidence that combination of amphotericin B with caspofungin may be active, but these results are preliminary.^139^

Certain Mucorales species have however variable susceptibilities to amphotericin, with MICs ranging from <1 to 100 μg/ml and treatment may be unsuccessful. Moreover, the drug may not reach the target because of tissue infarction and thrombosis.^140^ In order to increase oxygen concentration in tissues hyperbaric oxygen has been used as adjunctive therapy in limited studies, but evidence is insufficient to define the efficacy of this expensive intervention.^141^

Another option for the treatment of mucormycosis is the adjunctive use of iron-chelators deferasirox and deferiprone. A clinical trial is currently underway, testing the combination of deferasirox with liposomal amphotericin B.

Granulocyte colony-stimulating factor (G-CSF) and granulocyte-macrophage colony-stimulating factor (GM-CSF), G-CSF and GM-CSF are routinely given to neutropenic patients with mucormycosis or another invasive fungal disease. These two cytokines have also be used in a limited number of cases of mucormycosis in non-neutropenic patients as adjunctive treatment with favorable outcomes.^142^,^143^

Treating a patient’s underlying medical condition and reducing immunosuppression are essential to therapy. Rapid correction of metabolic abnormalities is mandatory in uncontrolled diabetes. Corticosteroids should be discontinued, if feasible, and other immunosuppressive drugs should be tapered as much as possible.

Diabetics may have a more favourable outcome than non-diabetics. An overall survival rate of 60–77% for rhino-orbito-cerebral mucormycosis has been reported in diabetics, versus 20–34% in non-diabetics.^144^,^145^ Combined surgical and medical therapy leads to reduced mortality rates compared to medical treatment alone.^6^,^146^

Despite the progress in the treatment of mucormycosis, many problems are yet unsolved and the mortality of the disease is still high.

## Conclusions:

Depending on the underlying condition of immunocompromised non- haematological patients, mucormycosis can manifest in various clinical forms: mostly as rhino-orbital or rhino-cerebral in diabetes patients, pulmonary infection in patients with malignancy or solid organ transplantation, disseminated infection in iron overloaded or deferoxamine treated patients, cerebral - with no sinus involvement - in ID users, gastrointestinal in premature infants, and cutaneous after direct inoculation in the immunocompetent.

Although there are many unresolved issues concerning the epidemiology, diagnosis and treatment of mucormycosis, advances have been made. Knowledge of the differential patterns of clinical presentation can raise the suspicion index for these rare but highly lethal infections. This is very important since prompt and appropriate management can reduce mortality and morbidity considerably.

## Figures and Tables

**Table 1:** Predisposing conditions for mucormycosis, pathogenetic mechanisms and clinical presentation.

**Predisposing conditions for mucormycosis**	**Pathogenetic Mechanism**	**Clinical presentation forms (in order of frequency)**
Haematological malignancies Haematopoietic stem cell transplantation (HSCT)	Prolonged neutropenia	1. Pulmonary, Sinus, 2. Cutaneous, 3. Sino-orbital
Diabetic ketoacidosis Uncontrolled diabetes mellitus	Impairment of neutrophil activation (functional neutropenia)/Fe usage by Zygomycetes for growth	1. Rhinocerebral, 2. Pulmonary, 3. Sino-orbital, 4.Cutaneous
Prolonged treatment with corticosteroids Autoimmune disease	Defects in macrophages and neutrophils, cortico- steroid induced diabetes, hypocomplementemia	1. Disseminated, 2. Renal, 3. Cutaneous, 4. Rhinocerebral, 5. Gastrointestinal
Solid organ transplantation (SOT)/graft versus host disease (GVHD)	Cellular Immune suppression, Corticosteroid induced diabetes	1. Pulmonary, 2. Sinus, 3. Cutaneous, 4. Rhinocerebral, 5. Disseminated
HIV infection/Intravenous illicit drug use	Injection of spores contained in drugs	1. Cerebral, 2. Cutaneous, 3. Renal, 3. Heart, 4. Rhinocerebral, 5. Disseminated
Iron overload, Iron/aluminium chelation therapy with deferoxamine (DFO)	Fe usage by Zygomycetes for growth Fe-DFO action as siderophore	1. Disseminated, 2. Pulmonary, 3. Rhinocerebral, 4. Cerebral, 5. Cutaneous, 6. Gastrointestinal
Skin or soft tissue breakdown burn/trauma/surgical wound/insect bite	Direct cutaneous inoculation with high number of spores	1. Cutaneous, 2. Pulmonary, 3. Sino-orbital, 4. Rhinocerebral, 5. Gastrointestinal.
Prolonged use of broad spectrum antifungal agents (voriconazole, itraconazole and caspofungin).	Breakthrough infection due to resistance in these agents	Sino-pulmonary
Miscellaneous Neonatal prematurity Malnourishment Prolonged use of broad-spectrum antimicrobial agents	Ingestion of spores Ingestion of spores Replacement of normal bacterial biota	1. Cutaneous, 2. Gastrointestinal, 3. Pulmonary, 4. Sino-orbital, 5. Rhinocerebral

**Table 2:** Percentages of mucormycosis cases according to underlying conditions. Data from 7 studies.

**Underlying conditions**	**Roden et al. [6] N=929**	**France Bitar et al. [12] 1997–2006 N=531**	**Italy Pagano et al. [13] 2004–2007 N=60**	**Belgium Saegeman et al. [14] 2000–2009 N=31**	**FUNGISCO PE Rüping et al. [15] 2006–2009 N=41**	ECMM/ISHAM Registry Skiada et al. [16] 2005–2010 N=230*	**India Chakrabarti et al. [17] 2000–2004 N=178**
**Haematol.**	21	17.3	61.7	77	63.4	55	1.1
**Diabetes Mellitus**	36	16.2	18	6.4	17.1	17	73.6
**SO Ca/T**	7	7.1	1.7	13	9.8	9	0.6
**DFO**	6					1	
**HIV**	2	4.9	1.7	3		2	
**AI/cortico**	1		3.3			7	
**Trauma/no underlying disease**	19	54.4	40	13		20	19.1

* Results from the 1^st^ ECMM study (ECMM/ISHAM Working group for a global registry for zygomycosis). Nineteen patients (8%) had more than one underlying disease, so the total is more than 100%.

N=number of cases, Haematol.=haematologic malignancies, myelodysplastic syndrome, aplastic anemia, haematopoietic stem cell transplantation (HSCT), SO Ca/T=solid organ cancer/transplantation, DFO=deferoxamine therapy, AI/cortico=autoimmune diseases/corticosteroid therapy.

**Table 3:** Clinical forms and relevant signs and symptoms

**Clinical forms**	**Symptoms and clinical manifestations**
Rhinocerebral, rhino-orbito-cerebral	Facial pain, headache, lethargy, loss of vision. Brownish, blood stained nasal discharge, black eschar on palate, chemosis, periorbital cellulitis, ophthalmoplegia, ptosis, proptosis, dysfunction of cranial nerves.
Pulmonary	*Nonspecific:* Chest pain, dyspnoea, haemoptysis
Cutaneous	Painful lesions, resemble ecthyma gangrenosum, cotton-like growth – “hairy pus”, necrotizing fasciitis.
Gastrointestinal	*Depend on site involved, non specific:* Abdominal pain, diarrhea, haematemesis, melena.
Disseminated	*Most frequent clinical syndromes:* Pneumonia, stroke, subarachnoid haemorrhage, brain abscess, cellulitis or gangrene.
Focal (can affect any organ)	*Protean manifestations according to involved organ:* Endocarditis, mediastinitis, peritonitis, osteomyelitis, pyelonephritis, otitis external, corneal infection

**Table 4:** Most frequently used antifungal agents to treat mucormycosis and entomophthoramycosis (adapted from ref. 123)

*Mucormycosis*
Amphotericin B deoxycholate (Fungizone®)
Amphotericin B cholesterol sulfate complex (Amphotec®, Amphocil®)
Liposomal amphotericin B (AmBisome®)
Amphotericin B lipid complex (Abelcet®)
Posaconazole (Naxofil®, as salvage therapy)
*Entomophthoramycosis*
Potassium iodide
Itraconazole
Ketoconazole
Miconazole
